# Therapy of hyperammonemia

**DOI:** 10.17179/excli2015-761

**Published:** 2015-12-22

**Authors:** Agata Widera

**Affiliations:** 1 Leibniz Research Centre for Working Environment and Human Factors at TU Dortmund (IfADo), Ardeystrasse 67, 44139 Dortmund, Germany

## ⁯

Recently, Ghallab and colleagues have identified a novel strategy to reduce hyperammonemia in mice (Ghallab et al., 2015[11]). The authors reduced blood ammonia concentrations by infusing a cocktail of glutamate dehydrogenase and its cofactors alpha-ketoglutarate and NADPH. This approach may be clinically relevant, because therapy of hyperammonemia is challenging (Levesque et al., 1999[17]; Enns et al., 2007[7]; Poh and Chang, 2012[20]). Currently hemodialysis is the treatment of choice for reducing strongly elevated blood ammonia concentrations (Ghallab et al., 2015[11]; Clay and Hainline, 2007[4]; Rajpoot and Gargus, 2004[21]). Therefore, infusion of glutamate dehydrogenase may represent a less invasive alternative.

At first glance, therapy of hyperammonemia with glutamate dehydrogenase seems counterintuitive. It is known that glutamate dehydrogenase generates ammonia in the periportal comportment of the liver lobule, which is then further metabolized by urea cycle enzymes (Ghallab et al., 2015[11]). Therefore, one may expect that glutamate dehydrogenase leads to an increase of ammonia instead of reducing its concentration. The hypothesis that glutamate dehydrogenase may detoxify ammonia came from a systems biology approach (Drasdo et al., 2014[6]). Recently, techniques of spatio-temporal modeling have been established (Drasdo, 2014[6][5]; Hoehme et al., 2010[15]). These techniques are based on reconstructions of tissue, where the position of each cell is known in a three dimensional space (Hammad et al., 2014[14]; Friebel et al., 2015[8]; Vartak et al., 2015[27]; Bartl et al., 2015[2]). Next, metabolic models can be integrated into the spatio-temporal model (Schliess et al., 2014[23]; Godoy et al., 2013[12]). Such models can be used to simulate, for example, the concentration of ammonia and associated metabolites in the liver vein (representing the liver 'outflow') for a given concentration in the portal vein (representing the 'inflow' of blood). Moreover, it can be simulated to which degree induction of liver damage compromises ammonia detoxification by the liver (Schliess et al., 2014[23]). Using such integrated spatio/temporal-metabolic models, Ghallab and colleagues have shown that the currently known metabolic pathways of ammonia metabolism by urea cycle and glutamine synthetase are not sufficient to explain the experimentally obtained data. Finally, modeling led to the prediction of an adaptive mechanism that occurs under conditions of toxic liver damage: glutamate dehydrogenase that normally supplies the urea cycle with ammonia switches its catalytic orientation to consume ammonia (Ghallab et al., 2015[11]).

Currently, hepatotoxicity represents an intensively studied topic (Campos et al., 2014[3]; Vitins et al., 2014[28]; Liu et al., 2014[18]; Messner et al., 2013[19]; Shimada et al., 2012[24]; Sumi et al., 2011[25]; Abdel-Bakhy et al., 2011[1]) and *in vitro* systems are frequently used in these studies (Grinberg et al., 2014[13]; Valente et al., 2015[26]; Ghallab et al., 2014[10][9]; Reif, 2014[22]; Ilkavets, 2013[16]). The study of Ghallab et al. shows that some adaptive mechanisms in response to toxicity may depend on complex features of tissue architecture and may be difficult to detect *in vivo*. For example, metabolic enzymes may adapt their flow rates or even switch their orientation. To nevertheless understand such complex situations, the novel techniques of mathematical modeling as introduced in the study of Ghallab et al. (2015[11]) represent a valuable tool.
